# Early trauma, attachment insecurity and dissociation as drivers of suicidal behaviour in borderline personality disorder

**DOI:** 10.1192/j.eurpsy.2026.11003

**Published:** 2026-07-31

**Authors:** D. Jelaga, M. Belous

**Affiliations:** Department of Mental Health, Medical Psychology and Psychotherapy, Nicolae Testemițanu State University of Medicine and Pharmacy, Chisinau, Moldova, Republic of

## Abstract

**Introduction:**

Borderline personality disorder (BPD) carries a high burden of suicidality. Clarifying how childhood adversity, attachment insecurity and dissociation relate to suicidal ideation, attempts and death in adults with BPD is essential for targeted prevention, yet evidence remains fragmented.

**Objectives:**

To quantify associations between early trauma, attachment insecurity and dissociation with suicidal ideation, attempts and death in adults with BPD; to examine subgroup differences (clinical vs community settings; comorbidity); and to explore mediation where reported.

**Methods:**

Comprehensive searches of MEDLINE/PubMed, Embase, PsycINFO and Web of Science in adults with BPD. Eligible designs: observational/longitudinal studies and systematic/narrative reviews; findings synthesised narratively (no meta-analysis). PRISMA flow: 1,196 records; 862 after deduplication; 147 full texts; 26 included (16 observational, 6 reviews, 4 longitudinal); 121 excluded (non-BPD 41%, <18 years 29%, no suicidality 21%, other 9%). Across included studies, mean age 29-36 years; 69-74% female; 78% clinical, 22% community.

**Results:**

Aggregated descriptively from included studies and recent reviews: adult BPD prevalences were ideation 74% (69-82%), attempts 56% (48-66%), deaths 4% (2-6%); inpatients typically showed +18-25 percentage points (pp) vs outpatients. Early trauma in 70-85% of patients; multiple exposures approximately +12-22 pp for attempts vs single/none; emotional abuse/neglect +15-28% for ideation. Attachment insecurity >70%; secure <30% and protective (attempt rates lower by 25-40%); where tested, secure attachment mediated ~10-20% of the trauma-suicidality association. Dissociation moderate-severe in 60-75%; associated with +18-30% ideation and with self-harm (non-suicidal self-injury); for attempts, differences vs low dissociation approximately +8-15 pp; longitudinal signals indicated elevated 6-12-month risk at higher dissociation. Subgroups (where reported): age <30 years +8-12 pp for attempts; comorbid major depressive disorder +20-30 pp.

**Conclusions:**

In BPD, early trauma, attachment insecurity and dissociation are key correlates of suicidal vulnerability, with measurable effects of ~10-30 pp on risk indicators. Clinical pathways should implement standardised screening (ACEs/CTQ, attachment measures, DES) and trauma-informed, attachment-focused and mentalisation-based elements alongside dialectical behaviour therapy (DBT).

**Disclosure of Interest:**

None Declared

